# D-Mannose Enhanced Immunomodulation of Periodontal Ligament Stem Cells via Inhibiting IL-6 Secretion

**DOI:** 10.1155/2018/7168231

**Published:** 2018-09-09

**Authors:** Lijia Guo, Yanan Hou, Liang Song, Siying Zhu, Feiran Lin, Yuxing Bai

**Affiliations:** ^1^Department of Orthodontics School of Stomatology, Capital Medical University, Beijing, China; ^2^Department of Orthodontics, Peking University School of Stomatology, The Third Dental Center, Beijing, China; ^3^Department of Stomatology, The Fifth People's Hospital of Shanghai, Fudan University, Shanghai, China; ^4^Laboratory of Tissue Regeneration and Immunology and Department of Periodontics, Beijing Key Laboratory of Tooth Regeneration and Function Reconstruction, School of Stomatology, Capital Medical University, Beijing, China

## Abstract

Periodontal ligament stem cell- (PDLSC-) mediated periodontal tissue regeneration has recently been proposed for the new therapeutic method to regenerate lost alveolar bone and periodontal ligament. It was reported that both autogenic and allogeneic PDLSCs could reconstruct damaged periodontal tissues but the regeneration effects were not consistent. The effective methods to improve the properties of PDLSCs should be further considered. In this study, we investigated if D-mannose could affect the immunomodulatory properties of hPDLSCs. After being pretreated with D-mannose, hPDLSCs could inhibit T cell proliferation and affect T cell differentiation into Treg cells. We found that less IL-6 could be detected in D-mannose-pretreated hPDLSCs. In the D-mannose pretreatment group, induced Treg cell number would decrease if increased IL-6 levels could be detected. Our data uncovered a previously unrecognized function of D-mannose to regulate the immunomodulatory function of PDLSCs and that IL-6 might play a key role in this process. The results provided a property method to improve PDLSC-based periodontal regeneration.

## 1. Introduction

Periodontitis, as one of the major oral infectious diseases, has high incidence in human. Periodontitis could cause damage to periodontal tissues, such as gingiva recession, attachment loss, alveolar bone loss, and teeth loss [1]. There is still no efficient therapy to recover the lost tissue. Stem cell-mediated periodontal tissue reconstruction is a promising strategy. Recently, periodontal ligament stem cells (PDLSCs) have received more and more attention in periodontal tissue reconstruction because of its multiple differentiation capacity and immunomodulation [2].

Recently, mesenchymal stem cells (MSCs) have been confirmed to have immunosuppressive and immunomodulatory properties and are extensively used to treat autoimmune diseases. Under the stimulation of inflammatory cytokines in microenvironment, MSCs inhibit the activation and proliferation of a variety of immune cells. Nevertheless, the role of MSCs on immune cells in different microenvironments remains partly unknown. More importantly, the diverse results suggested that the immunomodulatory functions of MSCs are involved in multiple factors. PDLSCs belong to one of various tooth-derived MSCs, which owned immunosuppressive abilities and mediate suppression by secreting inhibitory factors such as IFN*γ*, IDO, TGF*β*1, and HGF [3–6]. PDLSCs could inhibit T cell proliferation though PGE2 and promote T cell differentiation into Treg cells [7, 8]. When minipigs are transplanted with periodontal defects, PDLSCs could remodel the local immune microenvironment and obtain new tissue regeneration [9]. However, the detailed mechanisms were unknown, which caused unstable therapeutic outcomes in periodontal tissue regeneration.

Glucose plays critical roles in cell metabolism during energy generation and storage. At same time, glucose participates in some pathogenic processes, such as diabetes and obesity. D-Mannose is one of the important proteins in the glycosylation. The blood concentration of D-mannose is less than one-fiftieth of that of glucose. However, D-mannose has not received much attention. D-Mannose is a kind of C-2 epimer of glucose, which has been reported to as an effective therapy for urinary tract infections [10–15]. Currently, the function of T cell regulation of D-mannose has been found. D-Mannose could stimulate Treg differentiation by promoting TGF*β* signaling [16]. But whether D-mannose could affect immunomodulation of stem cell is still unknown. In this study, we cocultured T cells with D-mannose-pretreated human PDLSCs (hPDLSCs) to investigate the effect of D-mannose on hPDLSC immunomodulation function.

## 2. Materials and Method

### 2.1. Antibodies and Reagents

Purified anti-human CD3 (OKT3) and purified anti-human CD28 (CD28.2) were purchased from eBioscience. All fluorochrome-conjugated antibodies (anti-human CD4 (RPA-T4), anti-human CD45RA (HI100), anti-human CD25 (BC96), anti-human FoxP3 (PCH101), anti-human IFN*γ*, anti-human IL-4, anti-human IL-17, and anti-mouse IL-6 were from eBioscience. Recombinant human IL-2 (202-IL), human TGF*β*1 (240-B), and human latent TGF*β*1 (299-LT) were purchased from R&D Systems. Anti-TGF*β* (1D11.16.8), anti-CD25 (PC-61.5.3), and their isotype control antibodies (MOPC-21, HRPN) were from Bio X Cell. PGE2, TGF*β*, and IL-6 ELISA Ready-SET-Go! kits were purchased from eBioscience.

### 2.2. PDLSC Culture

PDLSCs were isolated and cultured from periodontal ligament tissues of periodontal healthy donors. The protocols for handling human tissues had been approved by the Research Ethical Committee of Capital Medical University. Healthy periodontal tissues from nine patients (age 18–36 years) were obtained. The periodontal ligament from the extracted teeth was separated from the surface of the roots and cut to small pieces. Then the small tissues were digested in 3 mg/ml collagenase type I (Worthington Biochemical, Freehold, NJ) and 4 mg/ml dispase (Roche Diagnostics, Basel, Switzerland) for 1 hour at 37°C. To get the single cells, all the cells were passed through a 70 *μ*m strainer (BD Labware, Franklin Lakes, NJ). Then about 1 × 10^5^ single cells were seeded into 10 cm culture dishes (Corning Costar, Cambridge, MA) with culture medium. The culture medium included *α*-modification of Eagle's medium (Gibco, Carlsbad, CA) and 10% fetal bovine serum (Equitech-Bio Inc., Kerrville, TX) supplemented with 100 mol/l ascorbic acid 2-phosphate (Wako Chemical, Tokyo), 2 mmol/l glutamine, 100 U/ml penicillin, and 100 *μ*g/ml streptomycin (Invitrogen, Carlsbad, CA). Then the cells were incubated at 37°C in 5% carbon dioxide. The colony cells were passed on day 14. PDLSCs in the study were three to four passages. All cells used in this study were at 3-4 passages. For each experiment, the same passages of hPDLSCs were used.

### 2.3. Surface Marker of PDLSCs after Glucose or D-Mannose Treatment

PDLSCs were cultured in “complete” glucose-free *α*-MEM culture medium supplemented with 25 mM D-mannose (M-hPDLSCs) or in the normal culture medium supplemented with 25 mM glucose (G-hPDLSCs). Three days later, surface marker expressions were analyzed by FACS staining. The treated PDLSCs were harvested with 0.25% trypsin, and cell suspensions (1.0 × 10^6^ cells) were incubated for 1 h at room temperature with monoclonal antibodies specific for CD90, CD45, CD44, CD73, and CD105 (BD Biosciences, Franklin Lakes, NJ, USA). Expression profiles of PDLSCs were analyzed by flow cytometry (BD Biosciences).

### 2.4. Osteogenic Differentiation Assay

PDLSCs were cultured in osteogenic medium. The inducing medium contained 2 mM *β*-glycerophosphate (Sigma-Aldrich, St. Louis, MO), 10 nM dexamethasone (Sigma-Aldrich, St Louis, MO), and 100 *μ*M L-ascorbic acid 2-phosphate (Wako Chemicals USA, Richmond, VA). The total protein was collected from induced PDLSCs after ten days. The gene expression levels of BGLAP and ALPL were assayed by RT-PCR analysis. The primer set for PCR included BGLAP (sense, 5-CGCTACCTGTATCAATGGCTGG-3, antisense, 5-CTCCTGAAAGCCGATGTGGTCA-3); ALPL (sense, 5-ATGGGATGGGTGTCTCCACA-3, antisense, 5-CCACGAAGGGGAACTTGTC-3); and GAPDH (sense, 5-AGCCGCATCTTCTTTTGCGTC-3, antisense, 5-TCATATTTGGCAGGTTTTTCT-3). To detect mineralized nodule formation, the cultured PDLSCs were stained with alizarin red after 4 weeks of induction.

### 2.5. Adipogenic Differentiation Assay

PDLSCs were cultured in adipogenic culture medium. The medium contained 500 *μ*M isobutylmethylxanthine (Sigma-Aldrich, St. Louis, MO), 500 nM hydrocortisone (Sigma-Aldrich, St. Louis, MO), 60 *μ*M indomethacin (Sigma-Aldrich, St. Louis, MO), 100 *μ*M L-ascorbic acid 2-phosphate, and 10 *μ*g/ml insulin (Sigma-Aldrich, St. Louis, MO). The gene expressions of peroxisome proliferator-activated receptor g (*PPAγG*) and FABP4 were analyzed via RT-PCR after adipogenic induction. The primer set for PCR included PPA*γ*G (sense, 5-CTCCTATTGACCCAGAAAGC-3, antisense, 5-GTAGAGCTGAGTCTTCTCAG-3); FABP4 (sense, 5-GTCCAGGCTGGAATGCAGTG-3, antisense, 5-CACACAGACGTACAGAGTGG-3); and GAPDH (sense, 5-AGCCGCATCTTCTTTTGCGTC-3, antisense, 5-TCATATTTGGCAGGTTTTTCT-3).

### 2.6. Alizarin Red Staining

After being induced for four weeks, the PDLSCs were fixed with 70% ethanol and stained with 2% alizarin red (Sigma-Aldrich). After being stained with alizarin red, the cells were destained for 30 min at room temperature with 10% cetylpyridinium chloride in 10 mM sodium phosphate and the calcium content was determined.

### 2.7. Oil Red O Staining

The cells were induced for 14 days in adipogenic medium and stained with Oil Red O (Sigma-Aldrich, St. Louis, MO). After being fixed with 4% paraformaldehyde, the cells were incubated with Oil Red O solution for 1 h. Then lipid droplets could be observed by microscopy.

### 2.8. Real-Time RT-PCR

Total RNA was derived from PDLSCs with an RNeasy mini kit (Qiagen). For real-time RT-PCR, cDNA was synthesized with a high-capacity cDNA reverse transcription kit (Applied Biosystems). Quantitative real-time PCR was performed using TaqMan gene expression assay kits (Applied Biosystems). The gene expression levels were normalized to the expression of *Hprt*.

### 2.9. Peripheral Blood Mononuclear Cells and CD4^+^ T Cells

Human peripheral blood mononuclear cells (PBMCs) from healthy volunteers were approved by the Research Ethical Committee of Capital Medical University. Blood samples were provided by the Capital Medical University School of Stomatology. All donors signed informed consent. Naive CD4^+^ T cells were purified by using Naive T Cell Isolation Kit II (Miltenyi Biotec). Then all the isolated cells were resuspended in T cell culture medium (Roswell Park Memorial Institute (RPMI))—1640 medium (GIBCO, Carlsbad, CA) with 10% FBS, 2 mmol/l glutamine, 20 mol/l HEPES, 100 U/ml penicillin, and 100 *μ*g/ml streptomycin (Invitrogen). To stimulate the naive CD4^+^ T cells, the cells were cultured in the anti-human CD3 (5 *μ*g/ml) precoated plate and soluble anti-human CD28 (2.5 *μ*g/ml) plus IL-2 (10 ng/ml). Three days after stimulation, the cells were analyzed by FACS staining.

### 2.10. T Cells Cocultured with PDLSCs

2 × 10^4^ human PDLSCs were seeded in 24-well plates in triplicate, and cells adhered to the plates and stayed overnight. Then the glucose- or D-mannose-pretreated PDLSCs were cocultured with CD4^+^ T cells (T + G-hPDLSCS/T + M-hPDLSCs) for 3 days in T cell culture medium stimulated with soluble anti-human CD3 (5 *μ*g/ml), anti-human CD28 (2.5 *μ*g/ml), and IL-2 (10 ng/ml).

### 2.11. T Cell Proliferation Assay

Activated T lymphocytes (1 × 10^6^/well) were cocultured with or without 0.2 × 10^6^ PDLSCs (pretreated with glucose or D-mannose) on 24-well multiplates with T cell-stimulated medium for 3 days. 1 × 10^4^ cells were incubated with 5 mM carboxyfluorescein succinimidyl ester (CFSE, Invitrogen) for 10 min. Five volumes of ice-cold medium were added to stop the staining process. After being washed three times, T cells were cultured for 72 h and analyzed by CFSE flow cytometry. A percentage of divided cells were analyzed by FSC Express 3.0 software.

### 2.12. *In Vitro* Th1 and Th2 Induction by PDLSCs

CD4^+^ T cells (1 × 10^6^/well) were cocultured with 0.2 × 10^6^ glucose- or D-mannose-pretreated PDLSCs on 24-well multiplates for 3 days in T cell culture medium stimulated with soluble anti-human CD3 (5 *μ*g/ml), soluble anti-human CD28 (2.5 *μ*g/ml), and IL-2 (10 ng/ml). After 3 days, cells in suspension were collected and detected Th1, Th2, Th17, and Treg via flow cytometry analysis. The concentrations of PGE2 and TGF*β*1 in supernatant were analyzed by ELISA Ready-SET-Go! kits (eBioscience) following the manufacturer's instructions. Gene expression of Nos2 and IDO1 in cocultured PDLSCs were analyzed by RT-PCR.

### 2.13. Flow Cytometry Analysis

Cells were incubated with PMA (10 ng/ml), ionomycin (250 ng/ml), and Golgi plug (1 : 1000 dilution; BD PharMingen) at 37°C for 4 h. For intracellular cytokine staining, cells were fixed with the fixation/permeabilization buffer solution (BD Biosciences). The collected T cells (1 × 10^6^) were stained with anti-CD4-FITC, anti-IFN*γ*-PE, anti-IL-6-PerCP, anti-IL-4-APC, and anti-CD25-PerCP. For intranuclear staining, the cells were continuously treated with fixation/permeabilization buffer solution (eBioscience) and stained with anti-FoxP3-PacBlue antibodies (each 1 mg/ml; eBioscience). Cells were carried out on a FACSCalibur, and data were analyzed with FlowJo software.

### 2.14. Statistical Analysis

All data were repeated in three to five independent experiments. Unless otherwise noted, statistical significance comparison was analyzed by two-tailed Student's *t*-test between two groups and by one-way ANOVA between more than two groups. Statistical analysis was performed with GraphPad Prism 6. *P* values less than 0.05 were determined statistically significant.

## 3. Results

### 3.1. D-Mannose-Pretreated PDLSCs Modulated T Cell Proliferation Better

**Figure 1:**
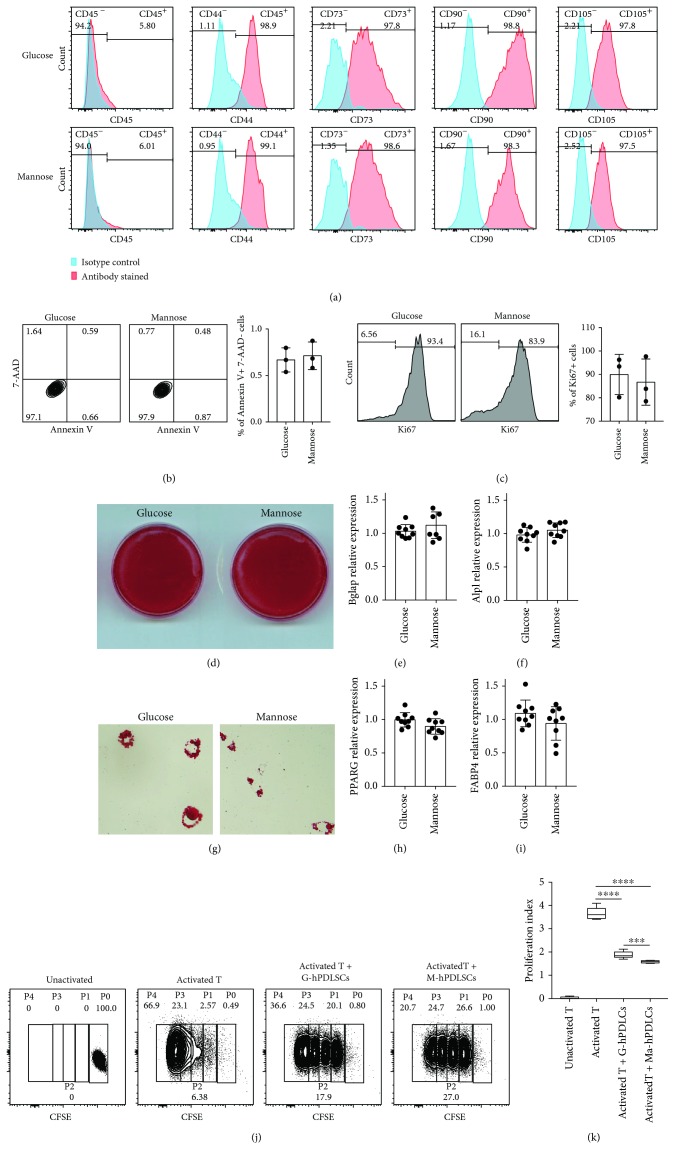
M-hPDLSCs have more inhibitory ability to proliferate T cells. (a) CD45, CD44, CD73, CD90, and CD105 have been detected by flow cytometry. There is no difference between M-hPDLSCs and G-hPDLSCs. (b, c) The apoptosis and proliferation of hPDLSCs between D-mannose and glucose treatment had no significant differences. (d–f) Osteogenic differentiation ability of M-hPDLSCs and G-PDLSCs has been detected. M-hPDLSCs and G-hPDLSCs were induced osteogenic differentiation. Alizarin red staining, BGLAP, and ALPL levels showed that M-PDLSCs have the same osteogenic differentiation ability as G-PDLSCs. (g–i) M-hPDLSCs and G-hPDLSCs were induced to adipogenic differentiation. Oil Red O staining, PPARG, and FABP4 levels showed that M-PDLSCs have the same adipogenic differentiation ability as G-hPDLSCs. (j, k) M-hPDLSCs and G-PDLSCs were cocultured with T cell to detect the effect of both on T cell proliferation. Both M-PDLSCs and G-PDLSCs could impair T cell proliferation. Compared with G-PDLSCs, M-hPDLSCs has more T cell proliferation inhibitory ability. Student's *t* test was used to analyze statistical significance. All error bars represent s.d. (*n* = 9). ^∗∗∗^*P* ≤ 0.001 and ^∗∗∗∗^*P* ≤ 0.0001.

In order to examine the effects of D-mannose on hPDLSCs, we used D-mannose or glucose medium to culture hPDLSCs. Surface makers of hPDLSCs, CD44, CD73, CD90, and CD105 were detected by flow cytometry Figure 1(a) and there was no difference between the two groups. There were no significant differences in apoptosis and proliferation of hPDLSCs between D-mannose and glucose treatment. We have added the data in (Figures 1(b) and 1(c)). We also found that D-mannose-pretreated hPDLSCs had no difference compared with the glucose-pretreated group on osteogenic differentiation and adipogenic differentiation potentials (Figures 1(d)–1(i)). Then we cocultured mannose- or glucose-pretreated hPDLSCs with T cells, and the results showed that mannose-pretreated PDLSCs (M-hPDLSCs) had more inhibitory ability to proliferate T cells than G-hPDLSCs (Figures 1(j) and 1(k)).

### 3.2. More Regulatory T Cells Were Generated When Cocultured with D-Mannose-Pretreated PDLSCs

**Figure 2:**
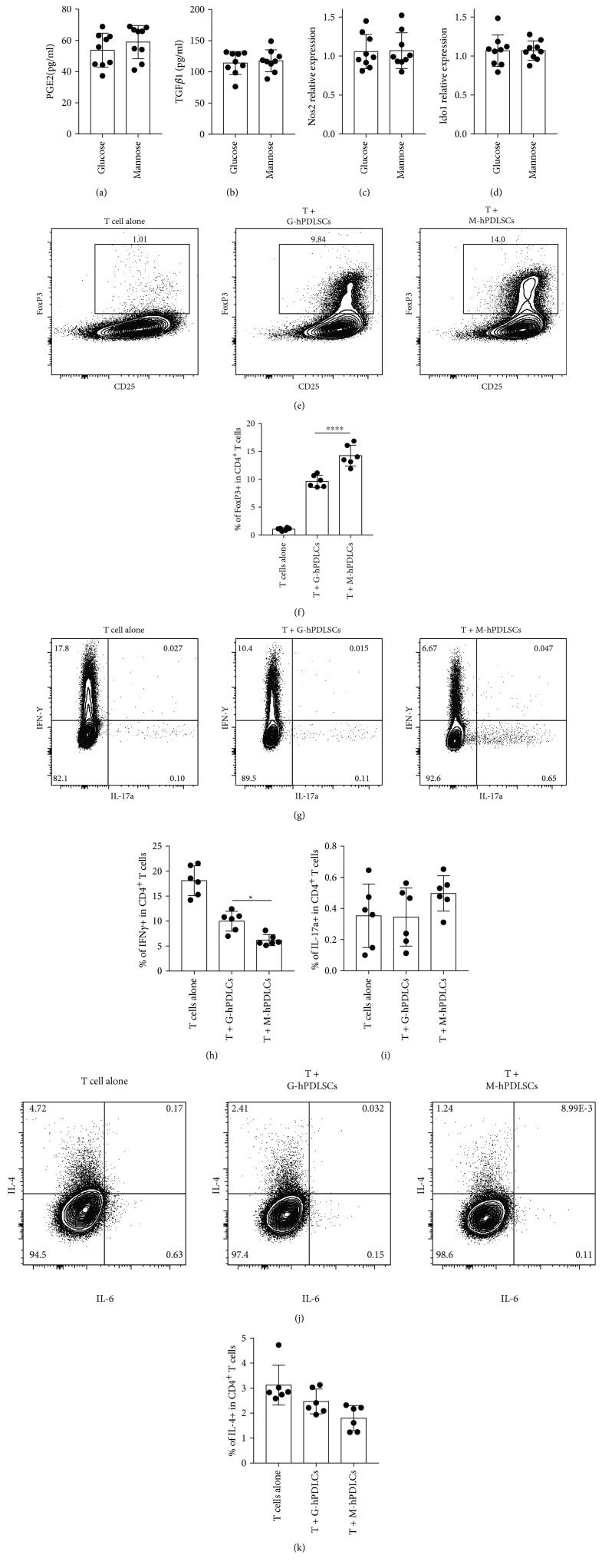
M-hPDLSCs induced more T cell differentiation into Tregs. (a, b) The results of ELISA showed that there was no difference in PGE2 and TGF*β*1 between T + M-hPDLSCs and T + G-hPDLSCs. (c, d) RT-PCR results showed that there is no difference in Nos2 and Ido1 between T + M-hPDLSCs and T + G-hPDLSCs. (e, f) FoxP3 have been detected by flow cytometry. Compared with T + G-hPDLSCs, more FoxP3 was detected in T + M-hPDLSCs. (g, h) IFN*γ* and IL-17 have been detected by flow cytometry. More IFN*γ* could be detected in T + M-hPDLSCs. There was no difference in IL-17 between T + G-hPDLSCs and T + M-hPDLSCs. (j, k) There was no difference in IL-4 between T + G-hPDLSCs and T + M-hPDLSCs. Student's *t*-test was used to analyze statistical significance. All error bars represent s.d. (*n* = 9). ^∗^*P* ≤ 0.05 and ^∗∗∗∗^*P* ≤ 0.0001.

To investigate how D-mannose affects hPDLSC immunomodulation function, T cells were cocultured with M-hPDLSCs or G-hPDLSCs. No difference was found in PGE2, TGF*β*1, Nos2, and IDO1 between both groups (Figures 2(a)–2(d)). However, more FoxP3+ T cells could be found in the T + M-hPDLSCs coculture system, suggesting that M-hPDLSCs induced more T cells differentiated to Tregs (Figures 2(e) and 2(f)). Furthermore, less Th1 could be found in the M-hPDLSC coculture system (Figures 2(g) and 2(h)) compared with G-hPDLSCs and there was no significant difference in Th17 (Figures 2(g) and 2(i)) or Th2 (Figures 2(j) and 2(k)) between these two groups.

### 3.3. D-Mannose-Pretreated hPDLSCs Secret Less IL-6 and Induced More Tregs

**Figure 3:**
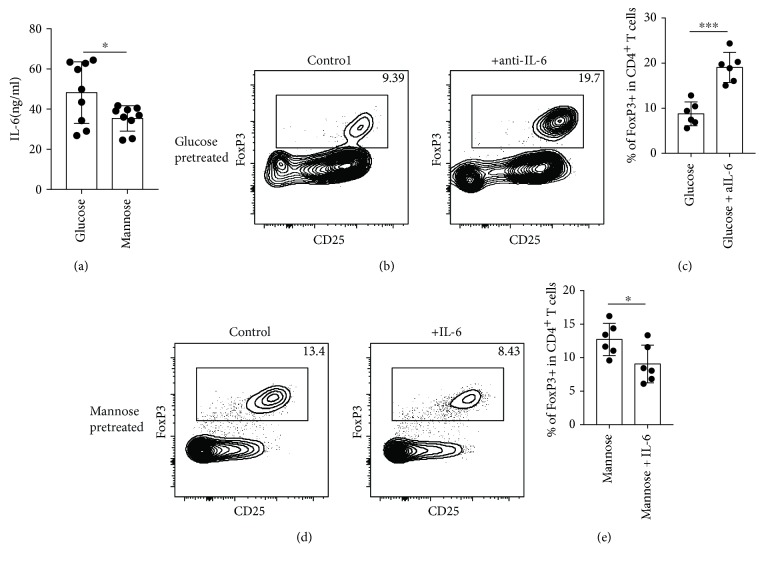
D-mannose inhibited IL-6 secretion of hPDLSCs to induce more T cell differentiation into Tregs. (a) Compared with T + G-hPDLSCs, less IL-6 could be detected in T + M-hPDLSCs. (b, c) Anti-IL-6 could increase the FoxP3 level in T + G-hPDLSCs. (d, e) Increased IL-6 could reduce the FoxP3 level in T + M-hPDLSCs. Student's *t*-test was used to analyze statistical significance. All error bars represent s.d. (*n* = 9). ^∗^*P* ≤ 0.05 and ^∗∗∗^*P* ≤ 0.001.

In order to know why the T cell M-hPDLSCs coculture system had more Tregs, we screened different cytokines (data not shown) and found that IL-6 was significantly lower in the T + M-hPDLSC coculture system. (Figure 3(a)). Interestingly, the number of Tregs increased after we neutralized IL-6 in T + G-hPDLSCs Figures 3(b) and 3(c) and decreased after we supply more IL-6 in the T + M-hPDLSC culture system (Figures 3(d) and 3(e)).

### 3.4. D-Mannose-Pretreated hPDLSCs Induced More Tregs *In Vivo* by Decreasing IL-6 Secretion

**Figure 4:**
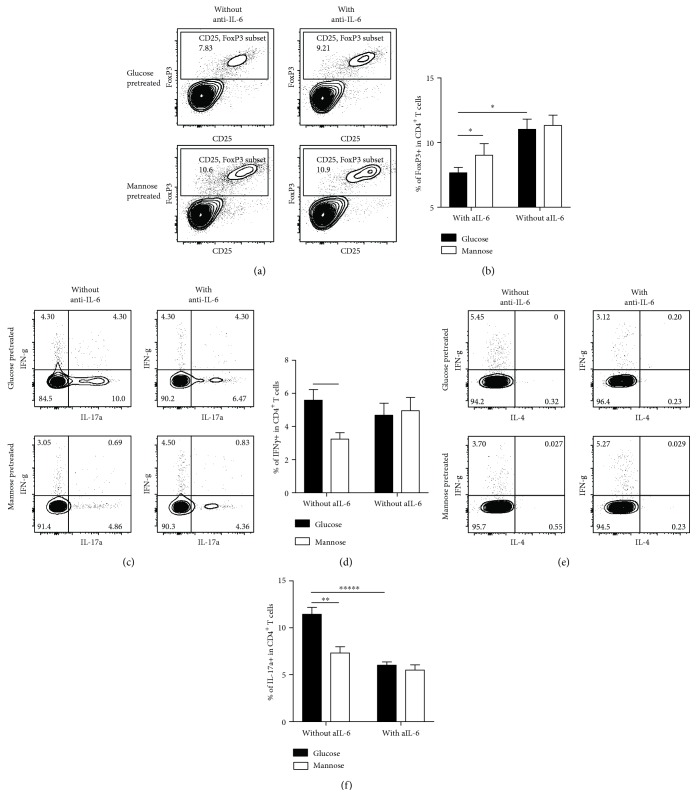
D-mannose inhibited IL-6 secretion of hPDLSCs to induce more T cell differentiation into Tregs in vivo. (a, b) IL-6 reduction could increase the number of FoxP3 and CD25 double positive cells in T + G-hPDLSC mixed cell-injected mice. (c, d) In vivo, compared with the T + G-hPDLSCs group, there was less Th1 cell in T + M-hPDLSC mixed cell-injected mice. (c, e) In vivo, IL-6 reduction could decrease the number of Th17 cell in T + G-hPDLSC mixed cell-injected mice. Student's *t*-test and one way ANOVA were used to analyze statistical significance. All error bars represent s.d. (*n* = 9). ^∗^*P* ≤ 0.05, ^∗∗^*P* ≤ 0.01, and ^∗∗∗∗∗^*P* ≤ 0.00001.

To verify previous results, we transplanted human T + G-hPDLSCs or human T + M-hPDLSC mixed cells with or without anti hIL-6 into nude mice. 2 days later, we extracted the spleen to examine T cells by flow cytometry. Without anti IL-6, the number of Tregs in the T + G-hPDLSCs group was much less than it in the T + M-hPDLSCs group. With anti hIL-6, the frequency of Tregs increased significantly in the T + G-hPDLSCs group (Figures 4(a) and 4(b)). These findings suggested that D-mannose could inhibit IL-6 in hPDLSCs to induce more Treg in vivo. On the other hand, more Th1 could be detected in the T + G-hPDLSCs group (Figures 4(c) and 4(d)) and IL-6 neutralizing could also reduce the number of Th17 (Figures 4(c) and 4(e)).

## 4. Discussion

Periodontitis is one of chronic infectious diseases destructing the alveolar bone and the supportive tissue of the teeth; it is also associated with a variety of systemic diseases such as diabetes, cardiovascular disease, and premature low birth weight [17–19]. The periodontal ligament comes from dental follicle, derived from neural crest cells, and PDLSCs play critical roles in periodontal ligament. PDLSCs could express the stem cell markers such as CD105, CD166, STRO-1, and CD146/MUC18 and own the properties of self-renewal and multipotency differentiation to osteo-like cells, adipocytes [20, 21]. PDLSCs participated into the whole process of periodontal tissue regeneration.

PDLSC-mediated periodontal tissue regeneration has been proposed for the development of new periodontal tissue. It is reported that both autogeneic and allogeneic PDLSCs could reconstruct damaged periodontal tissues but the regeneration effects were not consistent [22, 23]. Although lots of studies confirm that MSCs, such as PDLSCs, are generally thought to be poorly immunogenic, PDLSC transplantation in periodontal tissue engineering would not eliminate host immune rejection against the donor cells. Recently, many studies reported that MSC-mediated bone regeneration could be regulated by the host immune system, especially T lymphocytes. Proliferation of allogeneic lymphocytes could be elicited in both differentiated and undifferentiated MSCs [24–27]. On the other hand, it is well known that MSCs own immunomodulatory properties both *in vitro* and *in vivo*. MSCs could suppress the proliferation and differentiation of Th1 and Th17 cells [28, 29], as well as their productions (IFN*γ* and interleukin 17), while MSCs could enhance Th2 cells and the production (IL-4) [30, 31]. So, the crosstalk between immune cells and PDLSCs decided the tissue regeneration effects.

Previous studies found that the immunomodulatory properties of PDLSCs partially depended on soluble factors, which could be produced by PDLSCs after being stimulated by activated PBMNCs. When PDLSCs were cocultured with activated PBMNCs, PDLSCs could produce more TGF*β*1, indoleamine 2, 3-dioxygenase (IDO), and hepatocyte growth factor (HGF) [4]. hPDLSCs also could regulate the function of B cells. On the one hand, hPDLSCs could inhibit human B cell proliferation, differentiation, and chemotactic behavior. On the other hand, hPDLSCs could increase B cell viability by secreting interleukin-6. It was reported that the immunoregulatory capability of hPDLSCs to human B cells was through cell-to-cell contact manner, and programmed death-1 (PD-1) as well as its ligand (PD-L1) interaction was one of the critical ways in the process [32]. However, the interplay between host and transplanted PDLSCs during periodontal regeneration is unclear.

Recently, mannose was found as an important function in immune cell activity. D-Mannose could promote activation of the latent form of TGF*β* and enhance naive CD4^+^ T cell differentiation to Treg cells. However, the affection of D-mannose on the PDLSC characteristics was unclear. In this study, we found that D-mannose could affect hPDLSCs immunomodulation. hPDLSCs pretreated by D-mannose could inhibit T cell proliferation. As more results have shown, hPDLSCs pretreated by D-mannose could induce more T cell differentiation into Tregs and IL-6 played a key role in this process. Less IL-6 has been detected in T + M-hPDLSCs. When we increased the IL-6 level, less Treg cells could be detected in T + M-hPDLSCs; and when IL-6 was reduced, the number of Treg cells was increased in T + G-hPDLSCs. As we know, TGF*β* is an essential cytokine for inducing Foxp3+ Treg cells and enhanced TGF*β* signaling is an underlying mechanism. In our current study, TGF*β* levels between glucose- and D-mannose-pretreated groups had no significant difference.

IL-6 is a common cytokine and participates in almost every organ system's physiology. IL-6 could stimulate acute-phase responses, hematopoiesis, and immune reactions of the host to contribute to host defense. IL-6 plays an important role in the process of innate-acquired immune response. IL-6 could stimulate naïve CD4^+^ T cell differentiation [33]. It has been reported that IL-6 combined with TGF*β* is necessary for the process of naïve CD4^+^ T cell differentiation into Th17 [34]. IL-6 also could inhibit Treg differentiation induced by TGF*β* [35]. IL-6 plays a very important role in regulating Treg/Th17 balance. Breaking of the Treg/Th17 balance could be responsible for the collapse of immunological tolerance [36]. Further in vivo results also verified the important function of IL-6 in the process of D-mannose-regulating hPDLSC-stimulating Treg differentiation from T cells. It was reported that integrin *α*v*β*8 and reactive oxygen species (ROS) were essential for D-mannose-treated activation of TGF*β* T cells. However, how the detailed mechanism of D-mannose mediated IL-6 inhibition remains unknown.

In conclusion, our present results showed a new function of D-mannose on hPDLSC immunomodulation, exploring the important role of IL-6 in the process of D-mannose-regulating hPDLSC immunomodulation. Our findings provide more information for the basic immunological mechanisms of hexose sugars and provided the possible clinical applications of D-mannose.

## Data Availability

The data used to support the findings of this study are available from the corresponding author upon request.
